# Supplementary material for: Dietary Copper Deficiency Leads to Changes in Gene Expression Indicating an Increased Demand for NADH in the Prefrontal Cortex of the Rat’s Brain
Source: Int J Mol Sci. 2022 Jun 16;23(12):6706. doi: 10.3390/ijms23126706 (PMC9224161; doi:10.3390/ijms23126706)
Supplement: Supplementary file 1 [file ijms-23-06706-s001.zip › ijms-1772424-supplementary.pdf]

Table S1. Gene names and the results of real-time PCR analysis in all experimental groups, data presented as relative quantification ± standard deviation.

Gene name	Cu_{salt}			Cu_D			Cu_{MNP}		
	Mean ± RQ	W	P-value	Mean ± RQ	W	P-value	Mean ± RQ	W	P-value
Hif1a	1.024 ± 0.238	0.914	0.311	2.241 ± 0.191	0.955	0.728	1.149 ± 0.199	0.882	0.140
Myc	1.008 ± 0.130	0.950	0.675	0.812 ± 0.130	0.893	0.089	0.572 ± 0.066	0.989	0.773
Slc2a1	1.011 ± 0.161	0.868	0.096	0.880 ± 0.103	0.891	0.176	0.904 ± 0.129	0.956	0.735
Hk2	1.010 ± 0.151	0.947	0.627	1.692 ± 0.232	0.890	0.168	1.129 ± 0.258	0.905	0.247
Ldha	1.029 ± 0.237	0.878	0.123	0.903 ± 0.055	0.904	0.245	0.935 ± 0.276	0.868	0.098
Pc	1.005 ± 0.108	0.921	0.367	0.710 ± 0.114	0.927	0.417	0.798 ± 0.153	0.916	0.327
Mpc1	1.006 ± 0.109	0.924	0.388	1.200 ± 0.073	0.899	0.211	1.331 ± 0.130	0.938	0.526
Pdk4	1.010 ± 0.145	0.883	0.142	1.238 ± 0.086	0.895	0.190	1.273 ± 0.089	0.976	0.937
Mdh1	1.029 ± 0.253	0.875	0.115	1.247 ± 0.105	0.929	0.438	1.344 ± 0.396	0.909	0.276
Idh2	1.004 ± 0.096	0.886	0.152	1.099 ± 0.114	0.975	0.035	1.316 ± 0.359	0.889	0.167
Ppara	1.009 ± 0.143	0.950	0.671	1.535 ± 0.152	0.957	0.756	1.148 ± 0.144	0.878	0.079
Me1	1.011 ± 0.152	0.947	0.628	1.548 ± 0.122	0.932	0.467	1.053 ± 0.174	0.913	0.299
Cpt2	1.008 ± 0.133	0.975	0.935	1.570 ± 0.130	0.927	0.419	0.663 ± 0.121	0.860	0.077
Sod2	1.015 ± 0.179	0.937	0.519	2.343 ± 0.214	0.863	0.086	1.371 ± 0.347	0.848	0.093
Cat	1.007 ± 0.121	0.866	0.088	3.072 ± 0.346	0.854	0.064	1.321 ± 0.069	0.953	0.699
Gls	1.003 ± 0.085	0.985	0.986	3.201 ± 0.392	0.961	0.800	2.175 ± 0.609	0.897	0.204
Got2	1.024 ± 0.238	0.866	0.090	3.052 ± 0.195	0.946	0.619	1.843 ± 0.286	0.924	0.593

RQ, relative quantification; SD, standard deviation; W, the result of Shapiro-Wilk test; P-value, normality data.

Table S2. The symbols and names of the genes, oligonucleotide sequences, GenBank reference sequence accession numbers and the lengths of amplicon (bp).

Symbol of the gene	Name of the gene	Sequence (5′ → 3′)		NCBI Reference Sequence
		Forward	Reverse	
Slc2a1	Solute Carrier Family 2 Member 1	GCC TGA GAC CAG TTG AAA GC	GAG TGT CCG TGT CTT CAG CA	NM_138827.1
Hk2	Hexokinase 2	CTC CAT CCC ACA GGA GGT TA	TGA GGA GGA TGC TCT GGT CT	NM_012735.2
Pc	Pyruvate Carboxylase	GAG ATT GCC ATC CGA GTG TT	CTC CTT GGC CAC CTT AAT GA	NM_012744.2
Gls1	Kidney-type glutaminase, mitochondrial	CAC ACA CAC GGA TTT CTT GG	GCC GAA GCT GAC TTT GAA AC	NM_012569.2
Mpc1	Mitochondrial Pyruvate Carrier 1	ACT TTC GCC CTC TGT TGC TA	GCA CTG TCC CTT TCA AGA GC	NM_133561.1
Ldha	Lactate Dehydrogenase A	GGT GGT TGA CAG TGC ATA CG	AGG ATA CAT GGG ACG CTG AG	NM_017025.1
Pdk4	Pyruvate Dehydrogenase Kinase 4	CCT TTG GCT GGT TTT GGT TA	CAC CAG TCA TCA GCC TCA GA	NM_053551.1
Cpt2	Carnitine Palmitoyltransferase 2	TCC TCG ATC AAG ATG GGA AC	GAT CCT TCA TCG GGA AGT CA	NM_012930.1
Me1	Malic Enzyme 1	GCC CTG AAT ATG ATG CGT TT	CCT GGA ACA GCA CTG TCT GA	NM_012600.2

Ppara	Peroxisome Proliferator Activated Receptor Alpha	TCA CAC AAT GCA ATC CGT TT	GGC CTT GAC CTT GTT CAT GT	NM_013196.1
Cat	Catalase	ACA TGG TCT GGG ACT TCT GG	CAA GTT TTT GAT GCC CTG GT	NM_012520.2
Sod2	Superoxide Dismutase 2	CAC TGT GGC TGA GCT GTT GT	TCC AAG CAA TTC AAG CCT CT	NM_017051.2
Got2	Glutamic-Oxaloacetic Transaminase 2	ACC ATC CAC TGC CGT CTT AC	TCT TGA AGG CTT CGG TCA CT	NM_013177.2
Mdh1	Malate Dehydrogenase 1	GAA GCC CTC AAA GAC GAC AG	CGA CAG GGA ACG AGT AGA GC	NM_033235.2
Idh2	Isocitrate Dehydrogenase (NADP$^+$) 1	CAG TCT GAC ATC CTG GCT CA	AGA TGC TGG CAA TAG GGT TG	NM_001014161.1
Myc	MYC Proto-Oncogene, BHLH Transcription Factor	CGA GCT GAA GCG TAG CTT TT	CTC GCC GTT TCC TCA GTA AG	NM_012603.2
Hif1a	Hypoxia Inducible Factor 1 Subunit Alpha	TCA AGT CAG CAA CGT GGA AG	TAT CGA GGC TGT GTC GAC TG	NM_024359.1
Rpl32	Ribosomal Protein L32	AGA TTC AAG GGC CAG ATC CT	CGA TGG CTT TTC GGT TCT TA	NM_013226
Tbp	TATA box binding protein	CCT CTG AGA GCT CTG GGA TTG TA	GCC AAG ATT CAC GGT GGA TAC A	NM_001004198.1
